# Integrated Analysis of Bulk and Single-Cell RNA Sequencing Data Reveal a Novel Prognostic Signature of Combining Cuproptosis- and Ferroptosis-Related Genes in Hepatocellular Carcinoma

**DOI:** 10.3390/ijms26062779

**Published:** 2025-03-19

**Authors:** Hua Wei, Jiaxin Peng

**Affiliations:** 1 School of Resources and Environmental Science and Engineering, Hubei University of Science and Technology, Xianning 437100, China; 2 Research Center of Beidou, Industrial Development of Key Research Institute of Humanities and Social Sciences of Hubei Province, Hubei University of Science and Technology, Xianning 437100, China; 3 School of Computer Science, National Unversity of Defense Technology, Changsha 410073, China; jiaxinpeng@nudt.edu.cn

**Keywords:** hepatocellular carcinoma, cuproptosis- and ferroptosis-related genes, heterogeneity, prognosis, molecular docking

## Abstract

As a common malignancy, hepatocellular carcinoma (HCC) proliferation and metastasis could be promoted by ferroptosis and cuproptosis. In this study, we screened out the differentially expressed cuproptosis- and ferroptosis-related genes (CFRGs) and identified the 17 informative prognosis-associated genes. A CFRG scoring model was constructed based on the subtypes identified by consensus clustering analysis and principal component analysis (PCA). Furthermore, the immune profile, expression of immune checkpoint genes (ICGs) and drug susceptibility were also compared between the two CFRG score groups. The results showed that patients with a high CFRG score had higher survival probabilities. The correlation analysis suggested that CFRG scores were negatively correlated with activated CD4.T.cell. The expression patterns of thirty ICGs and the half-maximal inhibitory concentration (IC_50_) values of 128 drugs displayed significant differences between the two CFRG score groups. A statistically significant difference in the efficacy of sorafenib was found between the two CFRG score groups. Moreover, based on multivariate COX regression analysis and weighted gene co-expression network analysis (WGCNA), we screened DLAT and SLC2A1 as signature genes. Molecular docking analysis revealed that DLAT and SLC2A1 had a strong binding affinity toward camptothecin, rapamycin, dactolisib, and luminespib. The correlation between the CFRG score and single-cell characteristics was further explored. The study depended on our understanding of the biological function of CFRGs in HCC and provided new insights for developing treatment strategies.

## 1. Introduction

Accounting for 8.3% of cancer death rates globally [1,2] the mortality of liver cancer has escalated and is estimated to be more than 56.4% by 2040 [3]. Hepatocellular carcinoma (HCC) originated in hepatocytes, consisting of nearly 90% of primary liver cancers, is an aggressive cancer with growing morbidity and up to a mortality rate of 59 percent [3,4,5].

Despite advancements in diagnostic and treatment techniques leading to improved prognosis for early-stage HCC patients, the overall survival outcome is disappointing due to its high malignancy and propensity for easy metastasis and drug resistance [6,7]. The stage of the tumor has great influence on the prognosis. In addition, previous studies have suggested that the high heterogeneity of HCC tumors, largely due to genetic heterogeneity and different etiologies such as hepatitis viruses, alcohol, obesity, diabetes, smoking, etc., limits the availability of predictive biomarkers and complicates the individual-based treatment, resulting in undesirable clinical efficacy [4,8,9]. At present, liver transplantation, surgical resection, local ablation with radiofrequency, and locoregional therapies are the main therapeutic treatment modalities for early-stage cancer patients [10,11]. Additionally, a large number of new therapies like immunotherapy and molecular-targeted drugs have extended the survival period of some patients with HCC for all these years [12]. Thus, increasing attention has focused on developing more effective predictors to promote our comprehension of variables affecting HCC development and prognosis [13]. Molecular typing is of critical importance in formulating personalized treatment strategies for HCC patients [14]. However, the present HCC staging methods principally give attention to clinicopathologic features and are devoid of a thorough analysis of molecular features [15]. Therefore, it is crucial to identify a creative prognostic biomarkers or models on the basis of genetic status and molecular subtypes that could be used as both an effective therapeutic target and a robust prognostic biomarker to provide guidelines for clinical diagnosis and treatment of HCC patients.

At present, cuproptosis and ferroptosis are two types of regulated cell death [16] independent of apoptotic pathways. As the latest discovered type of regulated cell death, cuproptosis, occurring through the straight binding of copper to the lipid acylation metabolite of the tricarboxylic acid (TCA) cycle, was closely relevant to mitochondrial respiration and works via different mechanisms in comparison to previously discovered types of cell death [16]. The unusual polymerization of lipid acylated proteins and decrease in Fe-S cluster proteins, can be induced by the interaction effects of superfluous copper with lipid acylation components in the TCA cycle [16,17], which further lead to proteotoxic stress and subsequent cell death. Previous studies have revealed that regulating copper homeostasis in anticancer therapies may be a viable and prospective approaches [18,19]. Ferroptosis is crucial in cancer biology, pathogenesis, therapeutic targets, and treatment resistance, distinguished by an accumulation of iron-dependent lipid peroxides. Ferroptosis is susceptible to oleophilic antioxidants and can be initiated in the case of dysregulation of the mitochondrial TCA cycle [20,21]. Some studies have shown that the regulative mechanism of ferroptosis can also affect the sensitivity of HCC cells to targeted therapeutic strategies. Imbalanced ferroptosis is associated with the triggering of diverse diseases, such as ischemic organ damage, neurodegeneration, and cancers [22]. The induction of ferroptosis could inhibit tumor progression and deterioration of common therapeutic resistant cells, as well as boost immunotherapy efficiency [23]. Collectively, the two forms of cell deaths can be influenced by the inhibition of the TCA cycle and are closely associated with drug resistance, cell metabolism, and signaling pathways [24,25]. Recent bioinformatic analyses and experiment studies have confirmed an intricate link between ferroptosis and cuproptosis [26,27]; for example, in primary liver cancer, ferroptosis inducers such as sorafenib and erastin could promote cuproptosis in tumor cells as copper-dependent lipoprotein aggregated increasingly [28].

Therefore, the regulatory mechanisms related to cuproptosis with ferroptosis are anticipated to be new targets for HCC treatment. Previous studies have established the cuproptosis- and ferroptosis-related gene (CFRG) score system or cuproptosis- and ferroptosis-related gene signatures in LUAD or breast cancer [29,30]. However, there is lack of studies focusing on a prognosis model combining cuproptosis with ferroptosis integrating bulk and single-cell RNA sequencing in HCC. Herein, we innovatively linked cuproptosis with ferroptosis and thoroughly explored the functions of CFRGs in HCC. Subsequently, a CFRG score system was established based on CFRG clusters and gene clusters and validated in the International Cancer Genome Consortium (ICGC) database. Additionally, various analysis methods were applied to study the response of CFRG subtypes to immunotherapy and chemotherapy. Our findings indicated that the CFRG score predictive model is effective in forecasting prognosis, immunotherapy responses, and drug sensitivity for HCC patients in different subtypes, and it has potential to be a valuable prognostic indicator and provide new ideas for targeted therapy in HCC and facilitate personalized treatments.

## 2. Results

### 2.1. Landscape of Prognosis-Associated CFRGs

**Figure 1:**
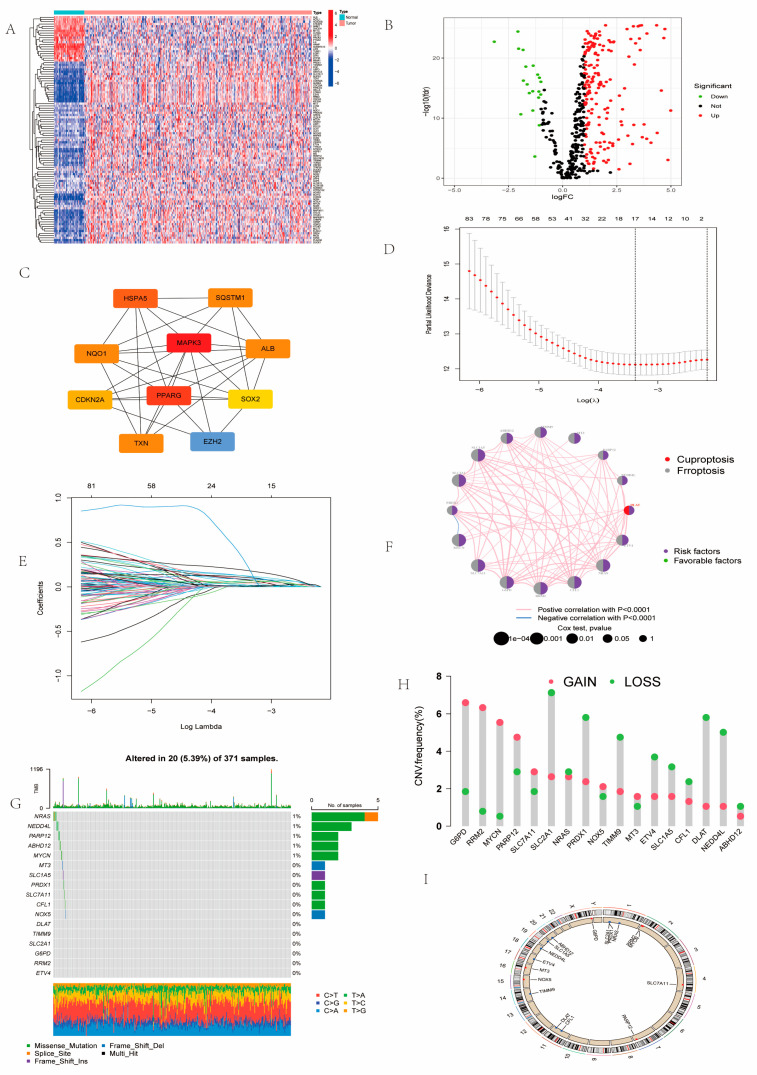
Gene alterations and expression analysis of prognostic-related CFRGs. (**A**) DEG heatmap based on the TCGA database. (**B**) Identified differentially expressed genes displayed by volcano map. (**C**) The top 10 genes identified by PPI network analysis of 97 prognostic genes. (**D**,**E**) 17 genes were identified by LASSO algorithm. (**F**,**G**) Prognostic networks and mutation landscape of 17 informative genes in HCC. (**H**,**I**) CNV frequencies of 17 informative genes and their location on 23 chromosomes in HCC.

Differential gene analysis among combining cuproptosis- with ferroptosis-related genes was conducted between HCC tumor and adjacent normal samples sourced from the Cancer Genome Atlas (TCGA) transcriptome dataset, and 203 differentially expressed genes (DEGs) were identified, with 20 genes downregulated and 183 genes upregulated (Figure 1A,B). Among these differential genes, we discovered that *TERT*, *MT3*, *NQO1*, *DUOX2*, *LCN2*, *SLC7A11*, *MIOX*, *SOX2*, *KIF20A*, *CDKN2A*, *MYCN*, *AQP8*, *CDCA3*, and *ADAM23* were significantly upregulated (log FC > 4). Next, we identified the genes related to prognosis using Kaplan-Meier and Cox regression analysis merging the Cancer Genome Atlas (TCGA) and GSE76427 dataset (Supplementary Table S1). The protein-protein interaction (PPI) network analysis showed that *MAPK3* has the highest number of adjacent nodes, which is a potential hub gene (Figure 1C). Next, LASSO regression analysis screened out 17 informative genes (Figure 1D,E). Prognostic network maps indicated *DLAT and SLC2A1* were risk factors among the prognosis-associated genes (Figure 1F). The informative genes were altered in 20 (5.39%) out of 371 samples (Figure 1G). From the results of copy number variation (CNV) analysis, *G6PD*, *RRM2*, *MYCN*, and *PARP12* showed significantly higher-frequency copy number gains, while *SLC2A1*, *PRDX1*, *TIMM9*, *DLAT* and *NEDD4L* displayed higher-frequency copy number deletions (Figure 1H). As the two most mutated genes, *SLC2A1* and *DLAT* were located on chromosomes 4 and 11, respectively (Figure 1I).

### 2.2. Identification of CFRG Subtypes, GSVA, ssGSEA, and Immune Score Analysis

**Figure 2:**
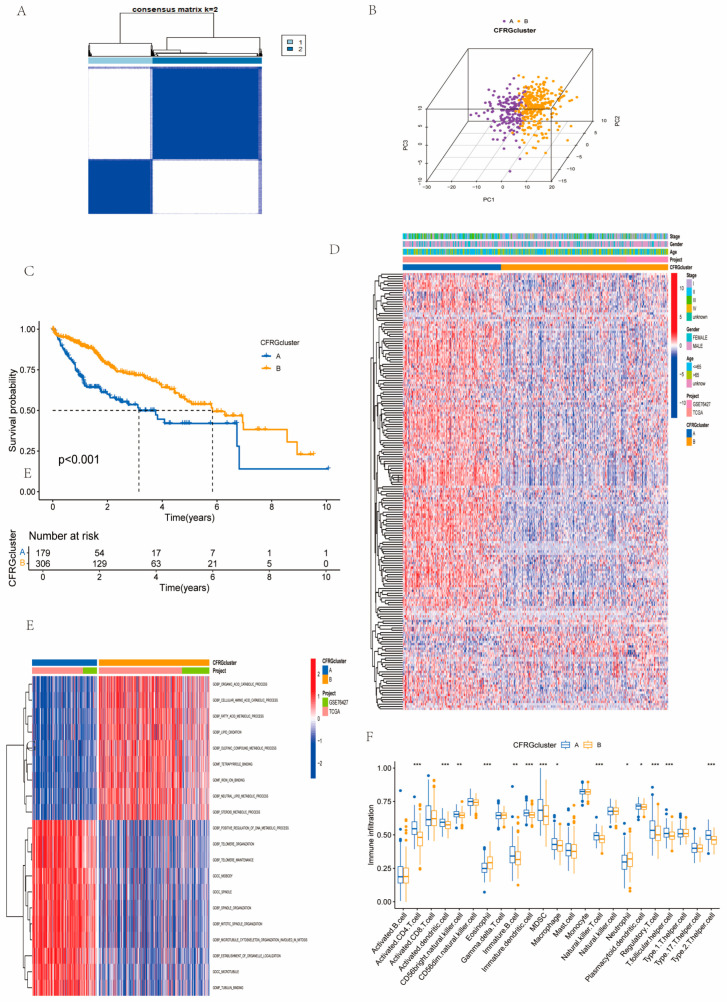
Clustering analysis of the CFRG clusters. (**A**) The optimal number of clusters based on the CDF curves and delta area of the cluster. (**B**) Two subtypes were distinguished according to PCA clustering. (**C**) Survival probability between two clusters. (**D**) A Complex heatmap displayed clinical correlations among the two CFRG clusters. (**E**) Comparison of CFRG clusters between A and B using the GSVA. (**F**) The differential analysis between immune cell infiltration for CFRG cluster A, B. * *p* < 0.05; ** *p* < 0.01; *** *p* < 0.001.

Since the optimal number of consistency matrix subtypes is k = 2, we classified the merged samples into two CFRG clusters (Figure 2A). The PCA revealed that two cuproptosis- and ferroptosis-related genes (CFRGs) clusters could be apparently distinguished according to the expression pattern of CFRGs (Figure 2B). Survival analysis revealed remarkable differences in the prognosis between two clusters, and cluster B showed better prognosis (Figure 2C). A comprehensive heatmap was established through integrating CFRG subtypes, clinical characteristics (age, sex, and tumor stage), and the expression levels of CFRGs (Figure 2D). The result revealed that most CFRGs were down-regulated in CFRG cluster B samples while being up-regulated in CFRG cluster A. Gene set variation analysis (GSVA) revealed that positive regulation of DNA_metabolic_process, telomere organization and maintenane were active in CFRG cluster A, while organic_acid_catabolic_process was active in cluster B (Figure 2E). The single-sample gene set enrichment analysis (ssGSEA) analysis revealed that the multiple T cells like activated CD4.T.cell, NK.T.cell, regulatory.T.cell, and T.follicular.helper.cell showed differential infiltration between the two CFRG clusters (Figure 2F).

### 2.3. Identification of Gene Subtypes and GO and KEGG Pathway Analysis

**Figure 3:**
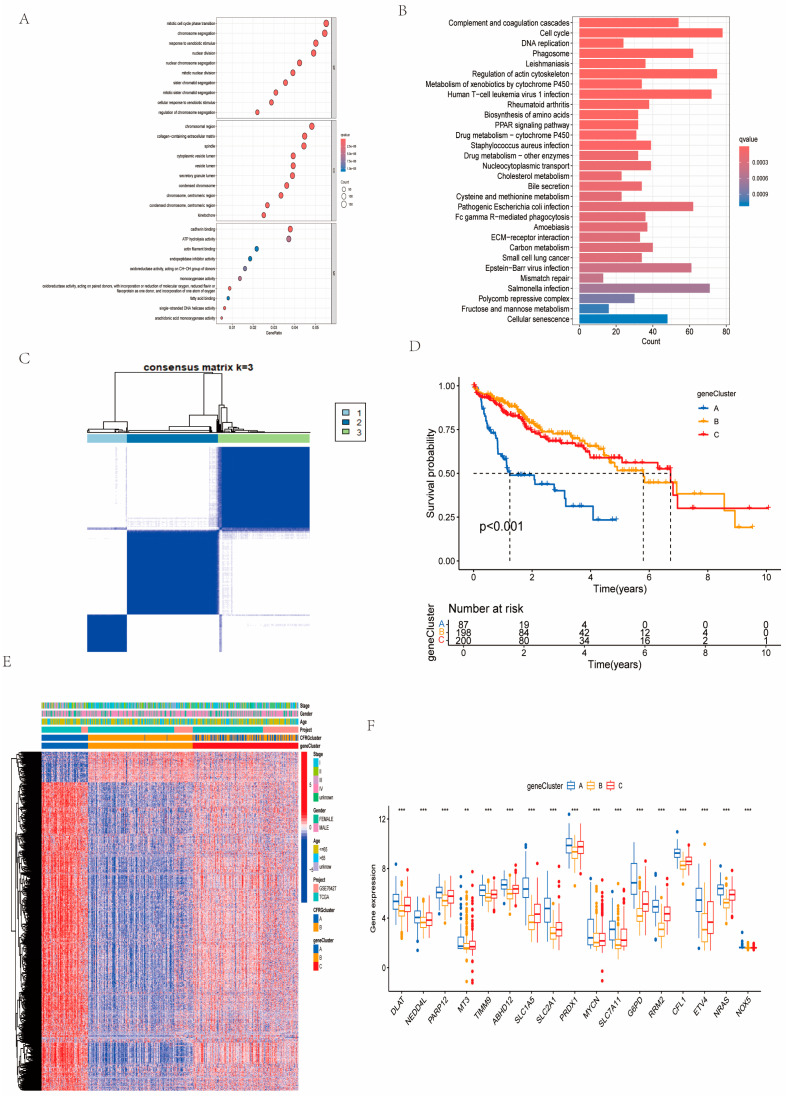
GO and KEGG enrichment analysis and clustering analysis of gene clusters. (**A**,**B**) GO and KEGG enrichment analysis of the intersecting genes. (**C**) Concordance matrix of identified gene cluster based on expression level of intersecting genes. (**D**) Survival probability of the gene clusters were compared using Kaplan–Meier survival method. (**E**) Expression patterns illustrated by the combined heatmap. (**F**) Expression pattern of 17 informative genes among the gene clusters. ** *p* < 0.01; *** *p* < 0.001.

According to Gene Ontology (GO) enrichment analysis, regarding biological processes (BPs), the intersecting genes between the two CFRG clusters were closely associated with the mitotic cell cycle phase transition and chromosome segregation; they were largely related to the chromosomal region, collagen-containing extracellular matrix, and spindle (CC). While related to molecular function (MF), they can influence cadherin binding and ATP hydrolysis activity, etc. (Figure 3A). Kyoto Encyclopedia of Genes and Genomes (KEGG) enrichment analysis suggested that these intersecting genes were largely relevant to complement and coagulation cascades, cell cycle, phagosomes, etc. (Figure 3B). According to a clustering analysis of the expression patterns of intersecting genes and clinical characteristics, all HCC cases were categorized into three distinct gene subtypes (Figure 3C). The survival probability indicated that patients in gene cluster A had the worst overall survival (OS) (Figure 3D). In addition, we constructed a comprehensive heatmap by integrating clinicopathological factors, CFRG clusters, and gene clusters (Figure 3E). The 17 informative genes were significantly expressed in the gene clusters (Figure 3F).

### 2.4. Construction of the CFRG Scoring Model

**Figure 4:**
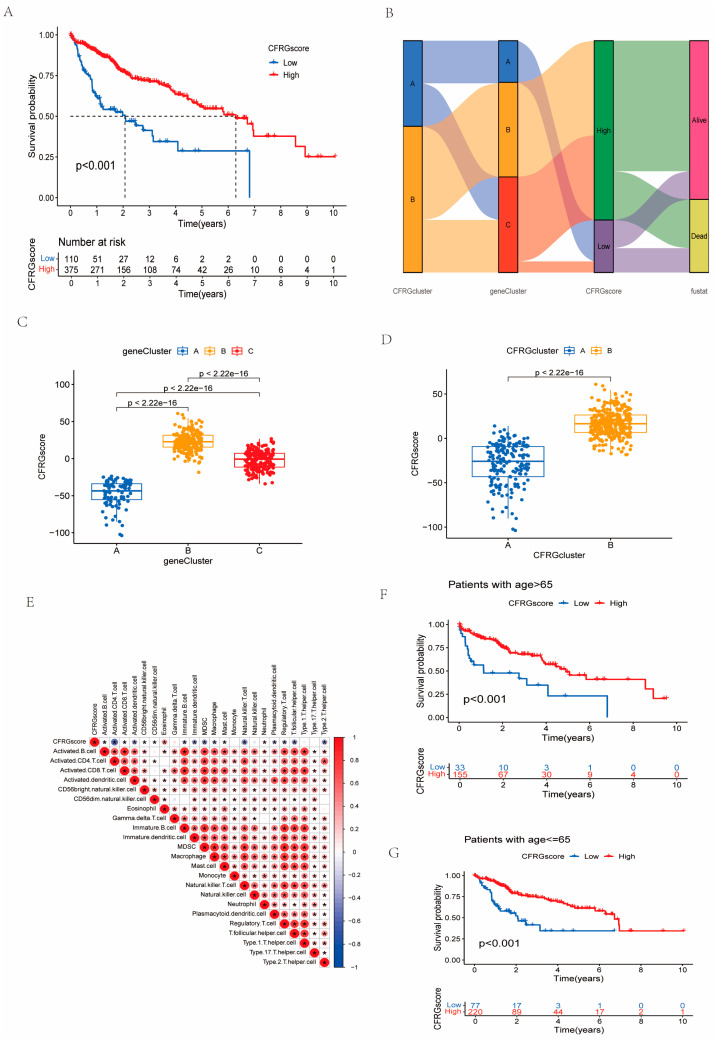
Construction of a CFRG score prognostic model of HCC. (**A**) Survival probability of two CFRG score groups were performed by the Kaplan–Meier survival method. (**B**) The construction process of the prognostic model was displayed by a Sankey diagram. (**C**,**D**) The differences in CFRG scores among the CFRG clusters and gene clusters. (**E**) The correlation between CFRG score and immune cells. (**F**,**G**) Differences in survival probability between groups of patients with age > 65 and age <=65.

According to PCA score analysis, these samples were grouped into high- and low-CFRG-score groups. The result of the survival difference analysis revealed there were distinct differences in the two CFRG score groups (*p* < 0.001) (Figure 4A). Furthermore, an alluvial diagram displayed the establishment process of the CFRG score (Figure 4B). The CFRG scores showed distinct differences in the two groups classified by survival states (*p* < 0.01). The proportion of alive patients were higher in the high-CFRG-score group (Supplementary Figure S1). Significant differences in the CFRG score value were observed across the CFRG clusters and gene clusters (Figure 4C,D). The correlation analysis suggested that CFRG scores were negatively correlated with activated CD4.T.cell, NKT.cell, T.follicular.helper.cell, and Type.2.T.helper.cell (Figure 4E). Importantly, the CFRG score value of cluster B in both the CFRG cluster and gene clusters were the highest. We observed that for HCC patients with age > 65 and <= 65 groups, the survival probability was significantly higher in the high-CFRG-score group (Figure 4F,G).

### 2.5. Difference Analysis of Drug Sensitivity, Immune State, Immune Checkpoint Inhibitors, and TIDE Score Between CFRG Score Groups

**Figure 5:**
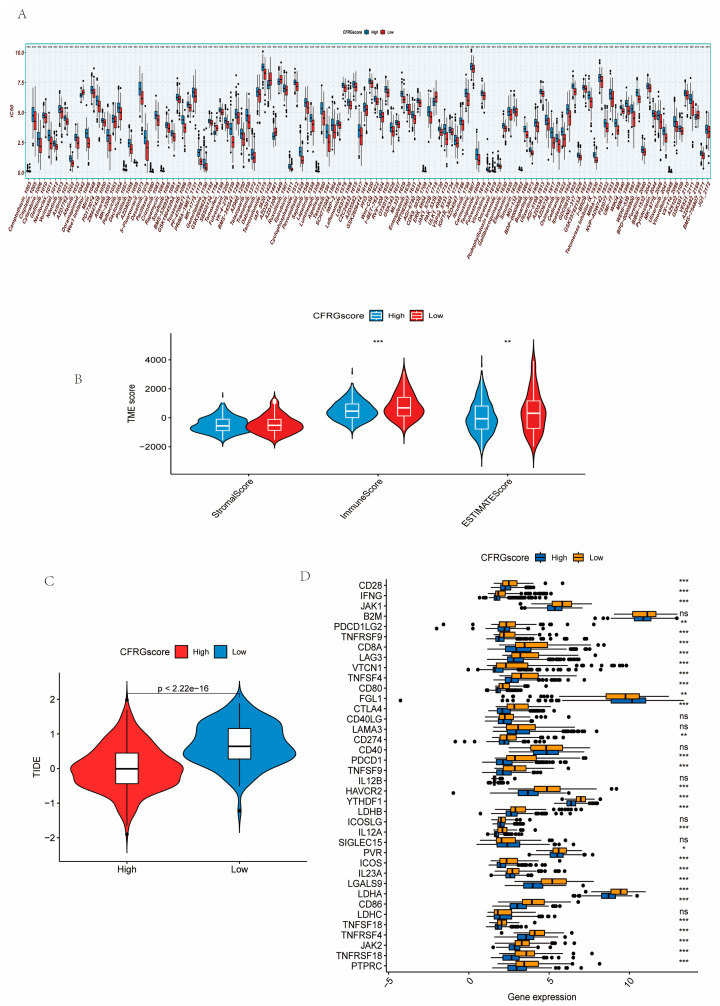
Testing of a CFRG score prognostic model of HCC. (**A**) Differences in drug sensitivity between two CFRG score groups. (**B**–**D**) TME state, TIDE score, and the expression levels of ICGs between the two CFRG score groups. * *p* < 0.05; ** *p* < 0.01; *** *p* < 0.001; ns, not statistically different.

Among a total of 198 drugs, the half-maximal inhibitory concentration (IC_50_) values of 128 drugs was markedly different between the two CFRG score groups (Figure 5A). The results revealed that the low-CFRG-score group had lower IC_50_ values (higher drug susceptibility) in most drugs. As shown by Figure 5B, the stromal score showed no statistical difference, while the immune and ESTIMATE scores were strikingly higher in the low-CFRG-score group. The tumor immune dysfunction and exclusion (TIDE) score was considerably higher in the low-CFRG-score group, indicating the high immune escape potential of these patients (Figure 5C). The result demonstrated that 30 immune checkpoint genes (ICGs) were significantly differently expressed between the two CFRG score groups and *FGL1* was remarkably expressed lower in the low-score group (Figure 5D).

### 2.6. Verification of the CFRG Scoring Model

**Figure 6:**
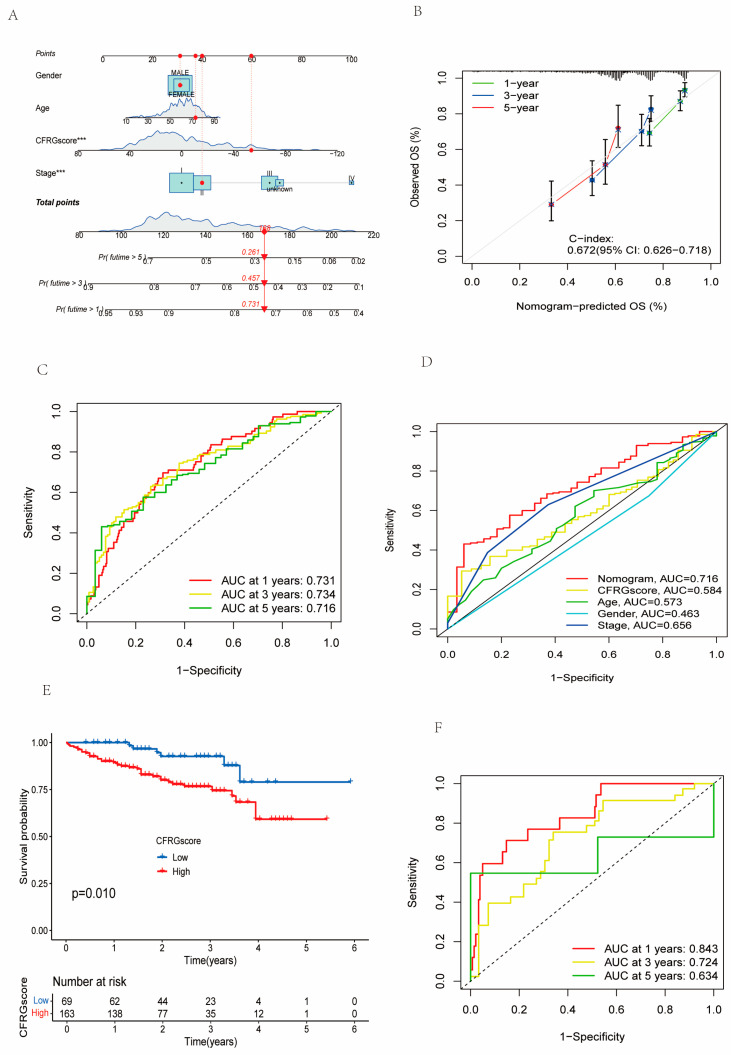
Verification of the CFRG score prognostic model of HCC. (**A**) The nomogram based on the CFRG score and clinical parameters (age, gender, and stage). (**B**) The accuracy of the nomogram revealed by calibration curves in the 1st, 3rd, and 5th years. (**C**) ROC curves for 1-, 3-, and 5-year overall survival probability of the nomogram. (**D**) Combined ROC curve including nomogram and CFRG score and clinical parameters (age, gender, and stage). (**E**,**F**) K-M curve and ROC curve for prediction of overall survival in the ICGC dataset. *** *p* < 0.001.

A nomogram was established to test the CFRG score hypothesis (Figure 6A), and its accuracy (C-index = 0.672) was further verified (Figure 6B). The area under curve (AUC) values were 0.731, 0.734 and 0.716 at 1, 3, and 5 years, respectively (Figure 6C), respectively. In the receiver operating characteristic (ROC) curve including nomograms, the CFRG score, and clinical factors (age, gender, and tumor stage), the AUC value of nomograms (AUC value = 0.716) is the highest (Figure 6D). As such, the nomogram could enhance the prognosis performance of the CFRG score model. The result of the ICGC dataset showed that survival probabilities of patients were significantly worse in the high-CFRG-score group (Figure 6E) and the AUC values were 0.834, 0.724 and 0.634 at 1, 3, and 5 years, respectively (Figure 6F).

### 2.7. Identification of Signature Genes

**Figure 7:**
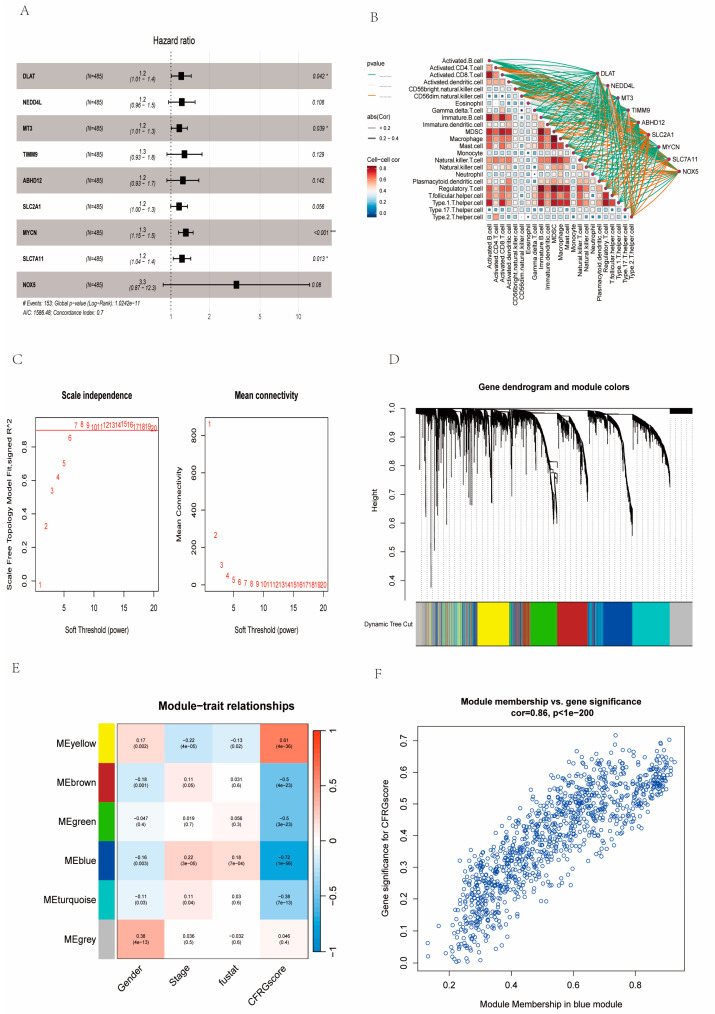
Identification of signature genes by WGCNA and CFRG score grouping. (**A**) Multivariate COX regression analysis of the informative genes. (**B**) The correlation analysis between these eight genes with immunological cells. (**C**) A map illustrating scale independence and mean connectivity with a soft threshold is presented. (**D**) The methods of dynamic tree cutting and dynamic merging are used to cluster the families among different modules. (**E**) Average correlation between various modules and gender, stage, survival state, and CFRG score. (**F**) The correlation between gene significance in the CFRG score and module membership in the turquoise module.* *p* < 0.05; *** *p* < 0.001.

Nine genes (*DLAT*, *NEDD4L*, *MT3*, *TIMM9*, *ABHD12*, *SLC2A1*, *MYCN*, *SLC7A11*, *NOX5*) were yielded through the multivariate COX regression analysis, and their correlation coefficients were calculated, of which *DLAT*, *MT3*, *MYCN*, and *SLC7A11* can be selected as independent prognostic factors (*p* < 0.05) (Figure 7A). Next, the correlation analysis showed that *SLC2A1* had a relative strong correlation with most cells (R = 0.2–0.4) (Figure 7B). Through a weighted gene co-expression network analysis (WGCNA) of co-expression modules with expression differences in tumor stage, sex, survival state, and CFRG score, a soft threshold of seven was selected using the “pickSoftThreshold” function (Figure 7C). Various gene modules were classified by the “cutreeDynamic” function and via the “mergeCloseModules” function. Similar modules were merged (Figure 7D). Based on the CFRG score, the module “turquoise” showed the highest correlation value (R = 0.86) (Figure 7E,F). Eventually, *DLAT* and *SLC2A1* were the signature genes screened out by the CFRG score module via WGCNA.

### 2.8. Molecular Docking Analysis

**Figure 8:**
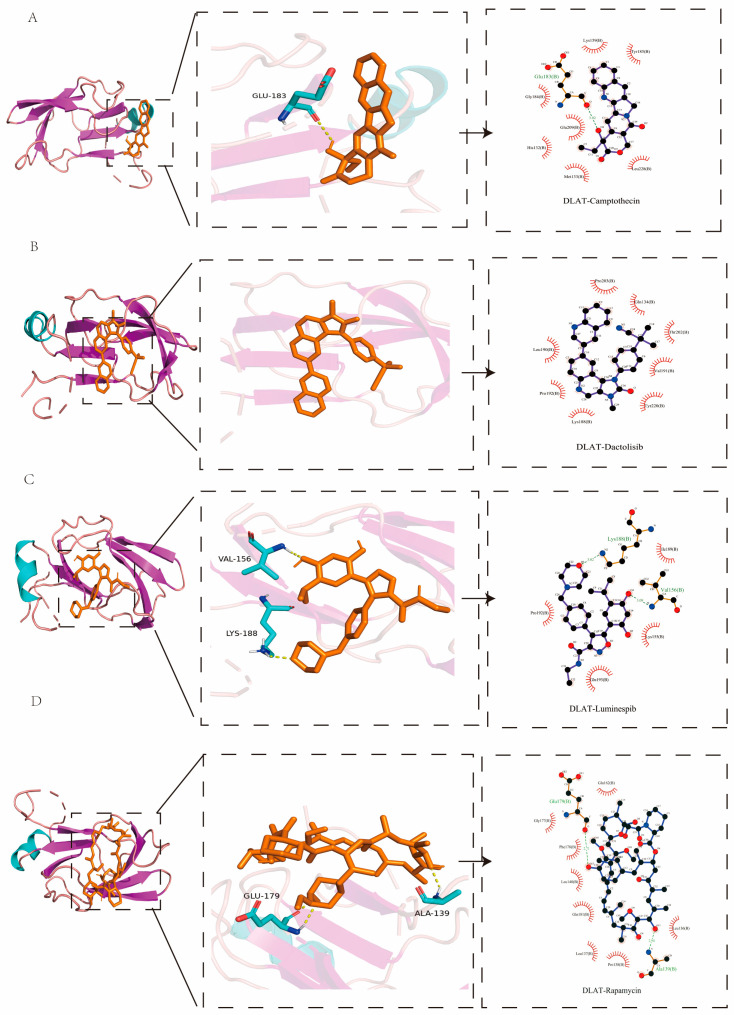
Three-dimensional (3D) and two-dimensional (2D) structure of the molecular docking results between DLAT and candidate drugs (**A**) camptothecin; (**B**) dactolisib; (**C**) luminespib; (**D**) rapamycin. Candidate drugs were displayed in brown, and molecular bonds (hydrogen) were marked in yellow (3D). Green dotted lines indicated hydrogen bonding (2D).

**Figure 9:**
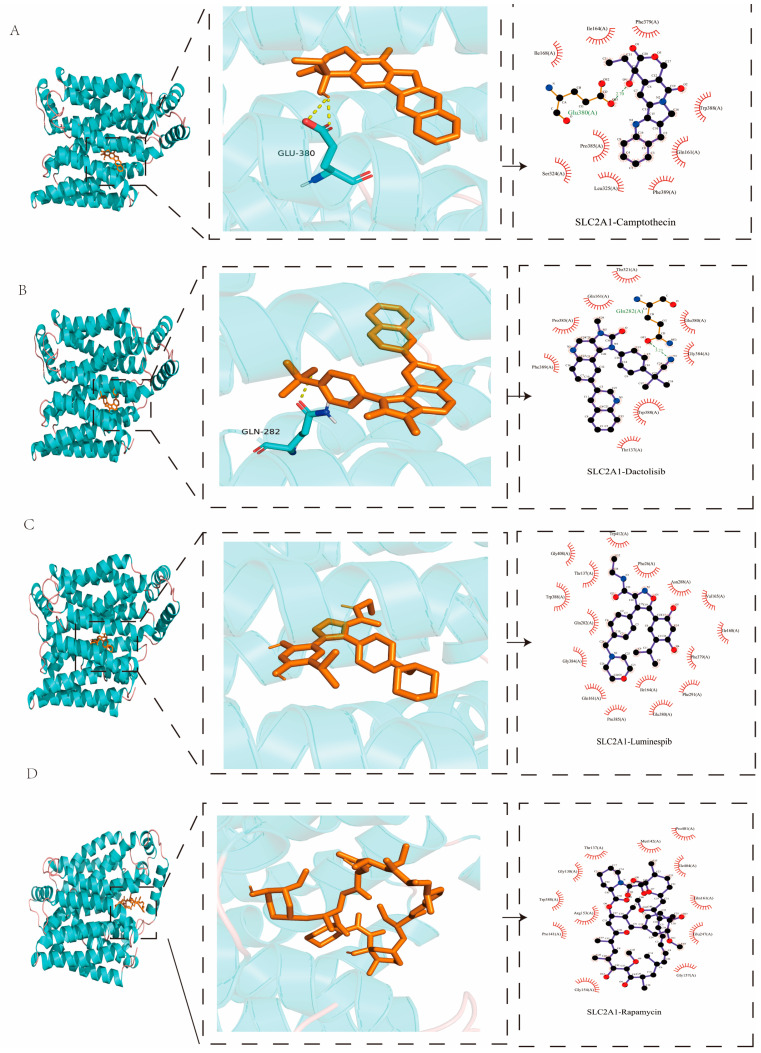
Three-dimensional (3D) and two-dimensional (2D) structure of the molecular docking results between SLC2A1 and candidate drugs (**A**) camptothecin; (**B**) dactolisib; (**C**) luminespib; (**D**) rapamycin. Candidate drugs were displayed in brown, and molecular bonds (hydrogen) were marked in yellow (3D). Green dotted lines indicated hydrogen bonding (2D).

We selected rapamycin, dactolisib, luminespib, and camptothecin with the highest drug susceptibility from Figure 5A to conduct molecular docking with DLAT and SLC2A1. From the molecular docking results shown in Figure 8, rapamycin and luminespib formed two hydrogen bonds with DLAT, while dactolisib and camptothecin formed one H-bond with SLC2A1 (Figure 9). As shown in Table 1, DLAT had the lowest binding energy toward camptothecin, followed by rapamycin, dactolisib, and luminespib, while SLC2A1 had the lowest binding energy toward dactolisib (−12.6 kcal/mol). In general, when the binding energy is less than −4 kcal/mol, it is recognized; the other factor is the hydrogen bond length, which is usually 1.5–3.5 A. The shorter hydrogen bond signified lower energy and a more stable system. The results showed that the four drugs had good binding properties with the two proteins.

### 2.9. Assessment of CFRG Score in the Single Cell Dataset

**Figure 10:**
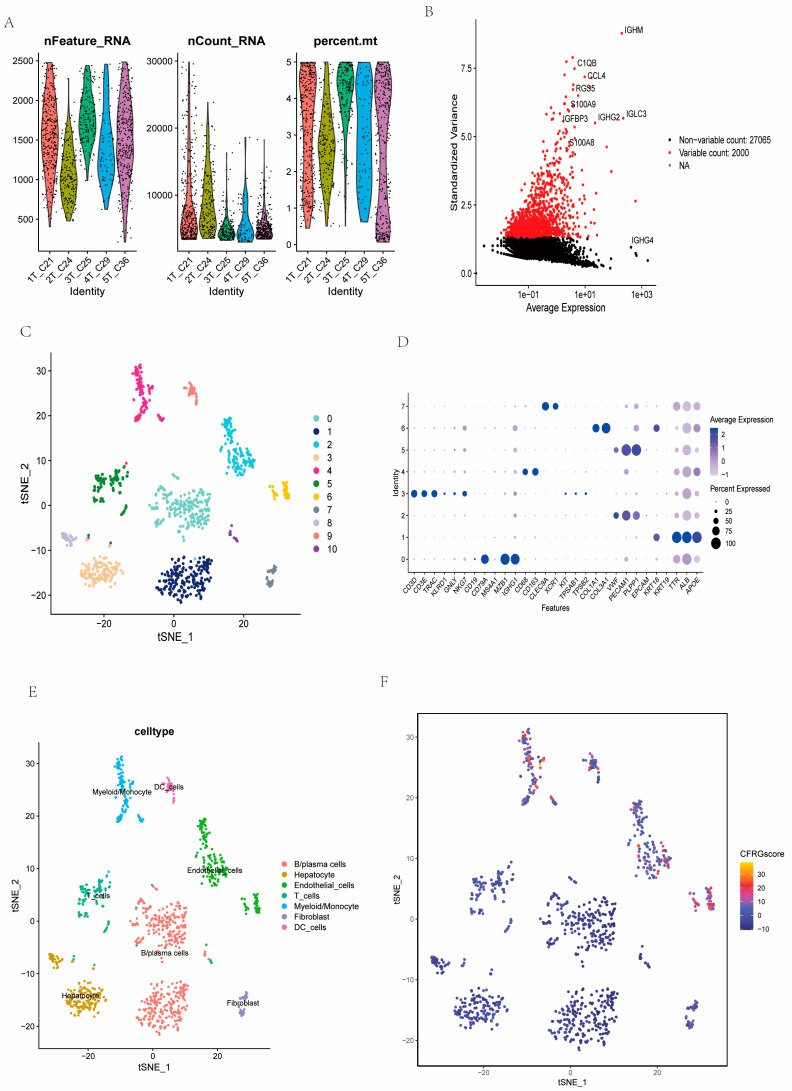
Estimation of CFRG score in GSE242889 single-cell clusters. (**A**) Quality control and data filtering of the five tumor samples. (**B**) The selected top 1500 high variable genes for downstream analysis with top 10 remarkable genes labeled. (**C**–**E**) The cell types showed by the t-SNE plot were annotated according to mark genes. (**F**) Assessment of the CFRG score in these cell types.

The single-cell dataset GSE242889 with five tumor samples were selected and then normalized after quality controls (Figure 10A). After dimensionality reduction conducted by PCA, we screened the top 1500 high variable genes and annotated the top 10 genes (Figure 10B), and 11 cell clusters were obtained eventually by the t-SNE algorithm. Via differential analysis across the whole cell clusters, these cell clusters were annotated into B/plasma cells, hepatocytes, T_cells, myeloids/monocytes, endothelial_cells, fibroblasts, and DC_cells, respectively (Figure 10C–E). The CFRG scores were higher in endothelial cells, DC_cells and myeloid/monocyte cells (Figure 10E,F).

**Figure 11:**
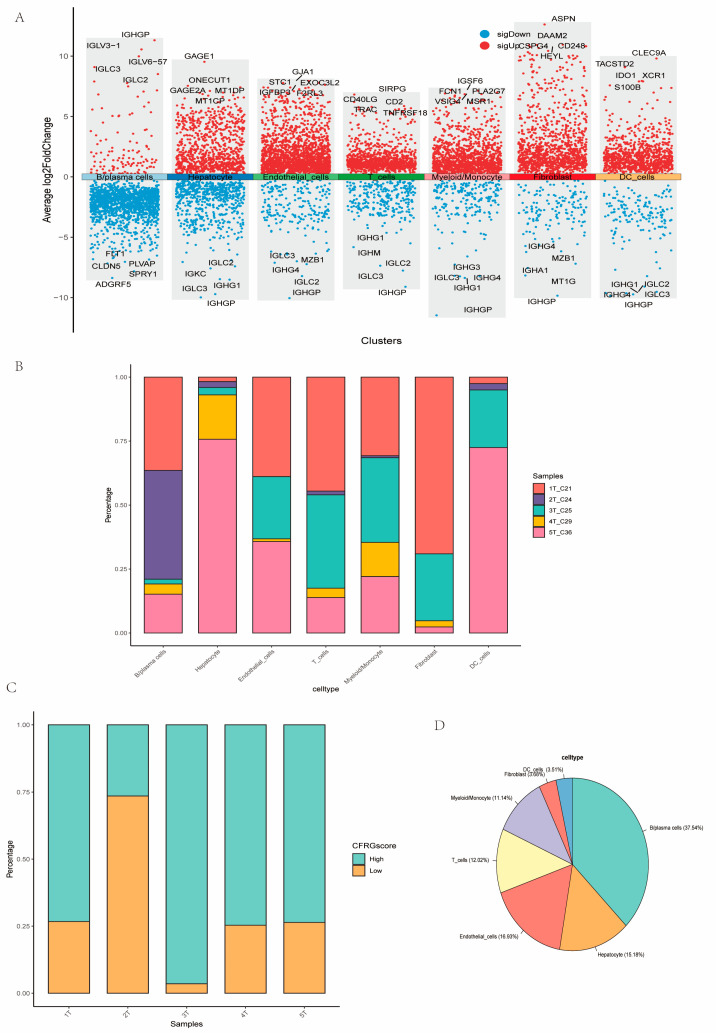
DEGs labeled among different types of cells and cell proportions in the samples. (**A**) The differential expression genes labeled among the cell types. (**B**) The percentage of different cell types in the samples (**C**,**D**) The proportion of the high- and low-CFRG-score group in the five samples.

We labeled the DEGs amog the multiple cells (Figure 11A). The multiple cell types accounted for different proportion in the five samples (Figure 11B). In addition, the high-CFRG-score group accounted for a majority of cells of the 3T sample, while the low-CFRG-score group comprised a predominant proportion of cells of the 2T sample (Figure 11C). B/plasma cells (37.54%) were predominant in the tumor samples, whereas there is a very small proportion of fibroblasts (3.68%) and DC_cells (3.51%) (Figure 11D).

**Figure 12:**
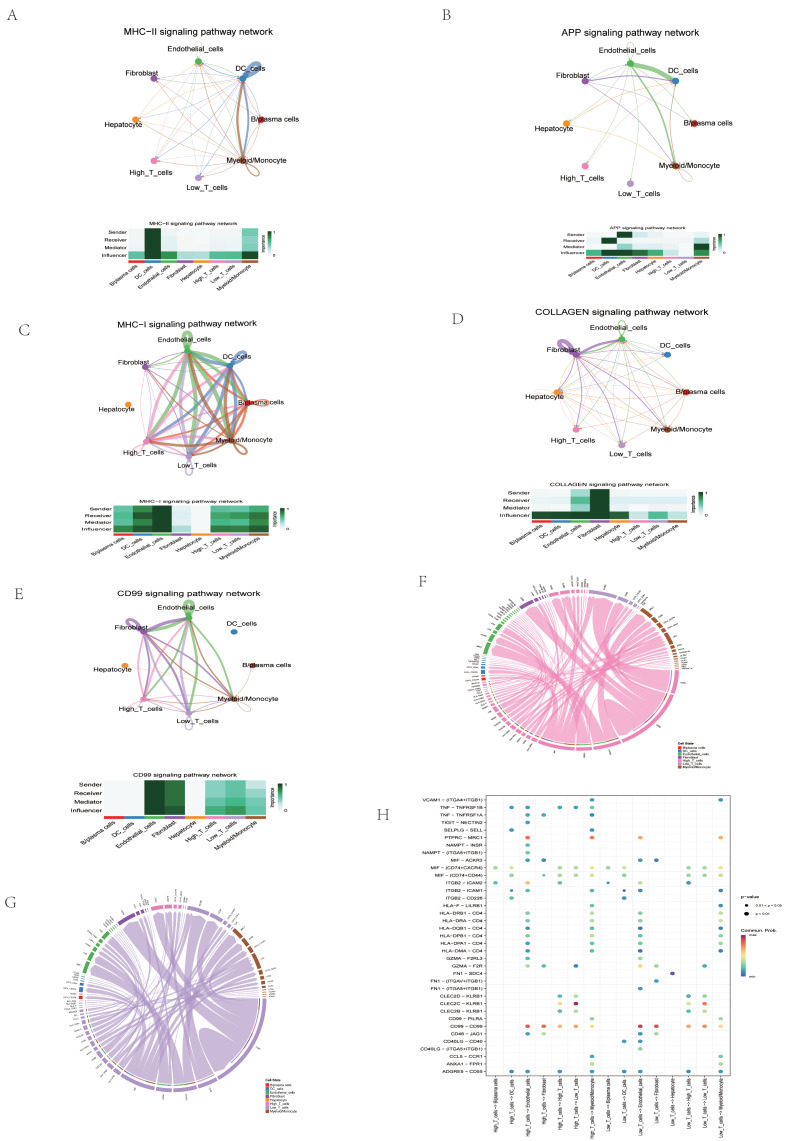
Intercellular communication difference between high- and low-CFRG-score T cells. (**A–E**) MHC−II, APP, MHC−I, COLLAGEN, and CD99 signaling pathway networks displayed by circus plots and the roles of various cell types in the pathway network illustrated by heatmaps. (**F**) The ligand–receptor interactions sourced from high-CFRG-score T cells. (**G**) The ligand–receptor interactions sourced from low-CFRG-score T cells. (**H**) Ligand–receptor pair difference.

Subsequently, T cells were grouped into high- and low-CFRG-score subgroups. Furthermore, the secreted signaling between T cells with other annotated cells were explored. T cells with high CFRG scores interact with more cell types in MHC−II and APP signaling pathway, whereas T cells with low CFRG scores interacted with more cell types that were considered as stronger influencers in the COLLAGEN signaling pathway network (Figure 12A–E). T cells with high and low CFRG scores showed multiple communication modes (Figure 12F,G). Furthermore, ligand–receptor pair analysis showed that high-CFRG-score T_cells preferentially sent signals to other cells by TNF−TNFRSF1B, TNF−TNFRSF1A, TIGIT−NECTIN2, SELPLG−SELL, NAMPT–INSR, and NAMPT−(ITGA5 + ITGB1) (Figure 12H). Collectively, high-CFRG-score T cells are likely to communicate more with other cells compared to low-CFRG-score T cells.

## 3. Discussion

Previous studies have demonstrated that the extensive variability and heterogeneity of HCC is a crucial factor contributing greatly to drug resistance and the recurrence of HCC [31,32]. Current studies indicated that copper exerts a critical role in anti-tumor therapy and tumor immunity [19,33], and ferroptosis can be induced by lipid peroxides or caused by some small molecule drugs such as erastin and sorafenib, and can also be supressed by inhibiting the mitochondrial TCA cycle activity [34,35].

The immunological classification of HCC offers inspiration for the treatment response and prognosis for HCC patients [36]. Previous studies have identified certain subgroups related to the recurrence or OS or cuproptosis-associated genes of HCC [13,37,38]. In this study, we innovatively screened out subtypes based on expression profiles and the molecular function of CFRGs in HCC. Survival difference analysis reveals there is distinct prognosis across two CFRG subtypes. Tumor-infiltrating immune cells play a pivotal role in immunotherapy, which revolutionized cancer treatment and prolonged survival time for tumor patients [39,40]. In our study, distinct differences were observed between two CFRG clusters concerning 14 immune cells, suggesting the two CFRG clusters exhibited distinct potential functions based on the ssGSEA algorithm.

The performance of the CFRG scoring system was evaluated through survival analysis, TME profiles, and drug sensitivity and proved to be effectively predictive. The tumor immune microenvironment plays a vital role in the biological responses and drug therapeutic effect of HCC [41]. We analyzed the immune, stromal, and estimate score between CFRG score groups, and the immune and estimate score variables displayed significant differences. This suggests that the CFRG score may be used to predict the TME structures. Then, a quantitative nomogram by merging the CFRG score with clinical factors (age, gender, and tumor stage) was developed, which greatly improved CFRG score performance and indicated its independent prognostic value. Regarding drug sensitivity, our study demonstrated that 128 drugs among 198 drugs obtained from the oncoPredict package showed distinctly different IC_50_ values between the two CFRG score groups, suggesting that our model probably predicts drug therapeutic efficacy. The multi-target kinase inhibitor sorafenib is an approved first-line systemic therapy for patients with advanced HCC [10,42]. A statistically significant difference in the efficacy of sorafenib was found between the two CFRG score groups in our study. Apart from sorafenib, it has been demonstrated that metronomic capecitabine as a second-line treatment has an anti-tumor effect and good safety for HCC patients failing first-line sorafenib therapy [43,44]. Other drugs such as lenvatinib [45], atezolizumab/bevacizumab [46], and cabozantinib [47] have also been explored and used for current sequential systemic treatment for HCC. According to the above analysis, the CFRG scoring system has potential to facilitate treatment decisions and strategies in personalized medicine for HCC, but its performance needs to be further tested in more effective drugs for HCC.

With the continuous emergence of new precise treatment for HCC, molecular targeted therapies and immune checkpoint blockade therapy have become notable therapeutic treatments [48,49]. Previous studies have shown that ferritin treatment can to some extent cause immune cells within the TME to trigger lipid peroxidation [50], and the function of these immune cells can be affected subsequently [51]. Using a ferroptosis inducer is a positive method to enhance the therapeutic efficiency of immune checkpoint inhibitors [51]. The expression levels of thirty ICGs including *PDCD1*, *LDHB*, *LDHA*, *LAG3*, *JAK2*, *IL23A*, *HAVCR2*, *CD8A*, and the TIDE scores revealed distinct difference in the two CFRG score groups. This indicates that our model has the potential to assess the expression profiles of immune checkpoints and the effect of immunotherapeutic efficiency. Noticeably, the strong negative correlation between CFRG scores and activated.CD4.T.cell/Type.2.T.helper.cell, which plays an important role in cancer immunology and facilitates the therapeutic effect in cancer patients, indicating that CFRG scores could be used as an indicator for the immunology classification of HCC patients. KEGG enrichment analysis suggested that intersecting genes were involved in complement and coagulation cascades, the cell cycle, phagosomes, and the regulation of the actin cytoskeleton, suggesting that our model has the potential for guiding the treatment strategies of HCC [52].

Based on the WGCNA algorithm and CFRG score, *DLAT* and *SLC2A1* were identified as potential signature genes. Previous studies have demonstrated that *DLAT* has the potential to be a promising prognostic biomarker and therapeutic indicator for the treatment of HCC [53,54]. *DLAT* expression was promoted by MELK through enhanced activity of PI3K/mTOR signaling, which led to stabilized mitochondrial function and eventually promoting the development of HCC [55]. Furthermore, the study by Kim et al. (2017) demonstrated that *SLC2A2* (GLUT2) is a novel prognostic factor for hepatocellular carcinoma [56]. The study by Peng et al. (2023) revealed that the Warburg effect inhibited through the SLC2A1 pathway could be prevented by dihydroartemisinin in HCC [57]. Through AutoDock’s molecular docking process, it was determined that DLAT and SLC2A1 possessed the high sensitivity to rapamycin and dactolisib, respectively. Collectively, these results provide theoretical bases for that *DLAT* and *SLC2A1* could be considered as potential therapeutic targets in the treatment of HCC.

In addition, the CFRG score was also evaluated between different cell clusters in the single-cell RNA-seq dataset. Among the annotated cell clusters, B/plasma cells were predominant in the samples. CFRG scores were higher in endothelial cells, DC_cells and myeloid/monocyte cells. Based on these results, we further identified the potential ligand–receptor interactions between and high-CFRG-score T_cells and other annotated cells. High-CFRG-score T_cells are likely to communicate more with other cells. Cuproptosis and ferroptosis may act as the bridge between HCC and immunocyte infiltration to affect the HCC progression.

The advantage of this study was establishing a scoring model using the PCA algorithm based on subgroups of HCC related to CFRGs integrating bulk and single-cell RNA sequencing data. Furthermore, this study has systematically and in-depth evaluated the indicators of the scoring systems and used diversified public datasets to establish and verify the model. Meanwhile, we have creatively applied the CFRG score system in single-cell datasets, which further prove the reliability of the prognostic model. Furthermore, the proteins encoded by potential signature genes *DLAT* and *SLC2A1 were* conducted molecular docking. The results of molecular dockings showed that the selected drugs had good binding properties with the two proteins, which suggested that DLAT and SLC2A1 may serve as potential therapeutic targets and can act as biomarkers, aiding in drug development and HCC classification. Nevertheless, there were also several limitations in this study. Firstly, the CFRGs collected in this study might not be comprehensive enough, since they are continuously being explored. Secondly, the expression of *DLAT* and *SLC2A1* at the transcriptome and protein levels was not experimentally verified and requires further studies. Thirdly, the relationship between cell subpopulation notes of T cells and the CFRG score can be further explored. Finally, since some drugs with good anti-tumor efficacy are not included in the oncoPredict package, more sophisticated algorithms need to be explored to test whether the CFRG scoring system could be applied to other effective anticancer drugs against HCC to improve clinical application.

## 4. Materials and Methods

### 4.1. Data Acquisition

**Figure 13:**
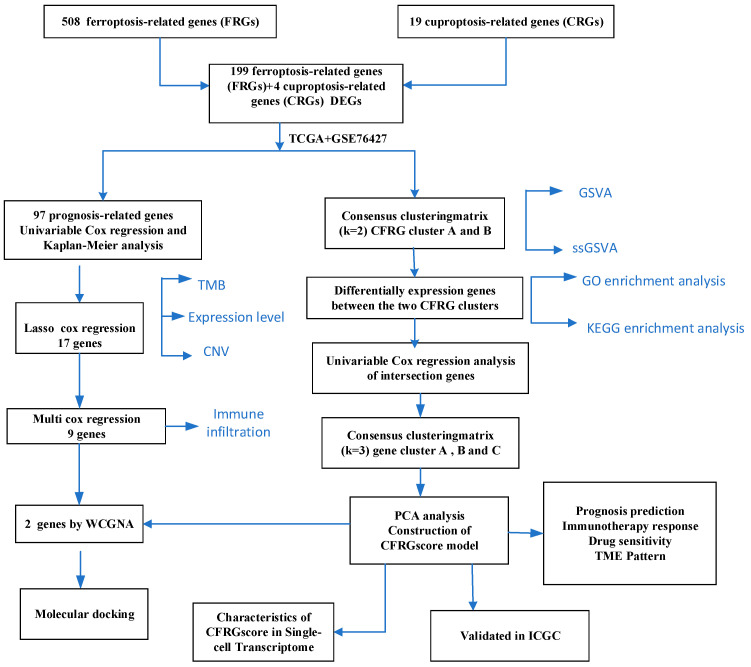
The flow diagram of this study.

The mRNA sequencing and matching clinical details were downloaded from TCGA and GSE76427 (Among HCC patients, 46% had HBV infection, and 54% had cirrhosis). Additionally, CFRG lists were collected from the previous studies and FerrDb website (http://www.zhounan.org/ferrdb/, accessed on 13 December 2023), respectively. Subsequently, the UCSC Xena server (https://xena.ucsc.edu/, accessed on 15 December 2023) was utilized to retrieve somatic mutation and copy number variation (CNV) data of liver hepatocellular carcinoma (LIHC). In addition, the LIRI-JP data were obtained from the ICGC (International Cancer Genome Consortium) database to validate the predictive model. Figure 13 presents the flow diagram of this study.

### 4.2. Gene Alterations and Expression Analysis and Prognostic-Related CFRGs

Using the limma package, DEGs between HCC and non-tumor samples were identified from count expression profiles with a threshold of |log2FC| ≥ 1 and FDR < 0.05. Volcano plot and heatmap showed the expression of DEGs using ggplot2 package. Using the STRING database (https://string-db.org/, accessed on 19 December 2024), we performed PPI network analysis on prognosis-related genes, of which the top 10 genes by node numbers were visualized by Cytoscape Software (3.9.1). LASSO regression analysis was applied to prognosis-related genes. The mutation frequency of these genes were calculated and visualized with waterfall plots using the “maftool” package (2.22.0).

### 4.3. Consensus Clustering Analysis, GSVA, and ssGSEA

The “ConsensusClusterPlus” R package was applied to establish CFRG clusters and intersection gene clusters [58]. The optimal cluster numbers were determined according to consensus clustering algorithm. The OS difference among these subgroups was identified by the “survival” R package (3.7.0). The gene set variation analysis (GSVA) and ssGSEA analysis was utilized to analyze the differences in biological pathways and immunological cell proportion across these subtypes. We also visualized clinical correlations among the CFRG clusters by heatmap. Subsequently, the GO and KEGG enrichment analysis of the intersecting genes between the CFRG clusters was carried out to find out the associated biological process, cellular components, and relevant pathways of the subtypes.

### 4.4. Establishment of CFRG Score Model

The CFRG scoring system was constructed by separating principal components (PCs) 1 and 2 through principal component analysis (PCA). Each patient’s CFRG score was determined by applying the following formula used in previous studies [13]:

CFRG score = Σ(PC1_i_) + Σ(PC2_i_)

where i represents the expression pattern of DEGs with the prognostic value of gene clusters. According to the optimal cutoff of the CFRG score, the whole samples were grouped into two subgroups. The predictive power of the scoring model was evaluated by time-dependent ROC curves. In addition, a nomogram using the CFRG score, age, gender, and clinical stage was constructed to enhance the prediction performance of the CFRG score model.

### 4.5. Assessment of Drug Sensitivity, Immunotherapy Efficacy, Stemness Scores, and Immune Checkpoint Expression Analysis

We evaluated tumor microenvironment (TME) including the immune, stromal, and ESTIMATE scores using estimation of stromal and immune cells in malignant tumors using expression data (ESTIMATE). We also performed correlation analysis of these scores with the survival of patients in HCC. Next, we screened out the sensitive drugs for cancer chemotherapy through the oncoPredict algorithm, calculating the 50% maximum inhibitory concentration (IC_50_) of different CFRG score groups. Furthermore, the TIDE Tool was applied to predict immunotherapeutic responses. The difference analysis of stemness scores and the expression patterns of immune checkpoints were also carried out between the two CFRG score groups to explore the effective immune checkpoint for tumor immunotherapy.

### 4.6. Signature Gene Identification Based on CFRG Score and WGCNA

We utilized the WGCNA to find pivotal genes based on the bulk RNA-seq dataset, with the aim of identifying gene modules that are highly correlated with CFRG scores. The genes intersected by the 17 genes and the gene modules with the highest CFRG scores were identified as signature genes.

### 4.7. Candidate Drug Prediction and Molecular Docking

Protein structures were downloaded from the Protein Data Bank (PDB) website (http://www.rcsb.org/, accessed on 9 December 2024). After being preprocessed with removing water molecules, adding hydrogen atoms, etc., the protein file was converted to a PDBQT format using AutoDock software (1.5.6). The structure of drug compounds with high sensitivity in the high CFRG score groups were obtained from PubChem (https://pubchem.ncbi.nlm.nih.gov/, accessed on 9 December 2024) database. After energy minimization processes, the file of pharmaceutical molecules was converted to a PDBQT format using AutoDock. Subsequently, the prepared molecules were performed molecular docking using AutoDock Vina. The binding energy between the protein and drugs, as well as the number of hydrogen bonds formed, are important criteria for evaluating the results of molecular docking. The lower the binding energy, the more hydrogen bonds formed, the more stable the binding will be, and the greater the likelihood that the target proteins will interact with the pharmaceutical molecules. The 3D and 2D maps of ligand-binding sites were displayed using PyMOL (3.1) and LigPlot+ (2.2.9) software.

### 4.8. scRNA-Seq Data Preprocessing and Evaluation of CFRG Score

The 5 tumor samples from the single-cell RNA-seq dataset GSE242889 were used to assess the expression profiles of genes and the CFRG score in the cell clusters. The scRNA-seq data were analyzed using the “Seurat” package. Using the CreateSeuratObject and “NormalizeData” function, the data underwent a quality control process (min.cells > 5, min. features > 50, percent. mt < 5%) and were then standardized. Next, we applied the “FindVariableFeatures” function to distinguish the top 1500 highly variable genes, which were then scaled and centered by the ScaleData function; then, dimensionality reduction was performed using the RunPCA function. Using the “FindNeighbors” and “FindClusters” functions, the most significant PCs were chosen for clustering the cells through the t-SNE (t-Distributed Stochastic Neighbor Embedding) algorithm. The “FindAllMakers” function was applied to marker DEGs for each cell cluster. Cell clusters were annotated based on the “SingleR” algorithm, previous literature, CellMarker (http://xteam.xbio.top/CellMarker/, accessed on 6 May 2024). The DEGs among cell clusters was remarked via the “jjVolcano” plot using the scRNAtoolVis package. In addition, signaling pathway networks between T_cells and other annotated cells were explored using the “Cellchat” package. We further analyzed differential ligand–receptor pairs between the two CFRG T_cells.

### 4.9. Statistical Analysis

Statistical analyses were performed using R (versions: R 4.4.1 and R 4.3.3) and Perl (v5.30.0). Differences between two groups were assessed using a t-test or Wilcoxon rank-signed test; for evaluating the difference among three subtypes, a one-way ANOVA test was conducted. Correlation analysis was conducted using either Spearman’s or Pearson’s test. p < 0.05 was considered statistically significant. * *p* < 0.05; ** *p* < 0.01; *** *p* < 0.001.

## 5. Conclusions

Taken together, this research innovatively combined cuproptosis and ferroptosis, identified subtypes of HCC, and ultimately established a CFRG scoring model for LIHC based on integrating bulk and single-cell sequencing data. The CFRG scoring model proved to be predictive of prognosis and immunotherapy response, indicative of drug sensitivity and independent of clinicopathological characteristics, which has the potential of being used for the assessment of chemotherapeutic drug sensitivity and predicting patients suited for immunotherapy. Furthermore, we performed a thorough analysis of the 17 informative CFRGs in HCC. The screened genes DLAT and SLC2A1 were considered as potential therapeutic targets, which is also verified by many other studies. Collectively, the study has depended on our understanding of the biological function of CFRGs in HCC and has presented some inspiration for making informed treatment strategies in personalized medicine for HCC patients.

## Figures and Tables

**Table 1:** The results of molecular docking between DLAT and SLC2A1 and the four selected drugs.

Protein	PDB ID	Targets	Affinity (kcal/mol)
DLAT	1Y8N	Camptothecin	−8.1
		Rapamycin	−7.6
		Dactolisib	−7.6
		Luminespib	−7.5
SLC2A1	4PYP	Dactolisib	−12.6
		Luminespib	−12.5
		Camptothecin	−10.8
		Rapamycin	−9.9

## Data Availability

The data presented in this study are available from TCGA database (https://portal.gdc.cancer.gov/, accessed on 19 November 2023), Gene Expression Omnibus (GEO) database (https://www.ncbi.nlm.nih.gov/geo/; GSE76427 dataset, accessed on 25 November 2023; GSE242889 dataset, accessed on 21 May 2024), and ICGC database (https://dcc.icgc.org/, accessed on 9 December 2023). These data were all available online in the public domain.

## Supplementary Materials

The following supporting information can be downloaded at: https://www.mdpi.com/article/10.3390/ijms26062779/s1.

## Abbreviations

HCC: hepatocellular carcinoma; CFRGs: cuproptosis- and ferroptosis-related genes; TME: tumor microenvironment; PCA: principal component analysis; ROC: receiver operating characteristic; scRNA-seq: single-cell RNA sequencing; GO: Gene Ontology; DEGs: differentially expressed genes; TCGA: The Cancer Genome Atlas; ICGC: International Cancer Genome Consortium; GSVA: geneset variation analysis; KEGG: Kyoto Encyclopedia of Genes and Genomes; ssGSEA: single-sample gene set enrichment analysis; TIDE: Tumor Immune Dysfunction and Exclusion; t-SNE: t-Distributed Stochastic Neighbor Embedding.
