# Supplementary material for: Integrated Analysis of Bulk and Single-Cell RNA Sequencing Data Reveal a Novel Prognostic Signature of Combining Cuproptosis- and Ferroptosis-Related Genes in Hepatocellular Carcinoma
Source: Int J Mol Sci. 2025 Mar 19;26(6):2779. doi: 10.3390/ijms26062779 (PMC11943219; doi:10.3390/ijms26062779)
Supplement: Supplementary file 1 [file ijms-26-02779-s001.zip › ijms-3449874-supplementary.pdf]

Supplementary Information:

Integrated analysis of bulk and single-cell RNA sequencing data reveals a novel prognostic signature related to cuproptosis and ferroptosis genes in hepatocellular carcinoma

Hua Wei[a*], Jiaxin Peng[b]

[a] School of Resources Environment Science and Engineering, Research Center of Beidou + Industrial Development of Key Research Institute of Humanities and Social Sciences of Hubei Province, Hubei University of Science and Technology, Xianning Hubei, 437100, China

[b]School of Computer Science, National Unversity of Defense Technology, Changsha, Hunan, 410073, China

A

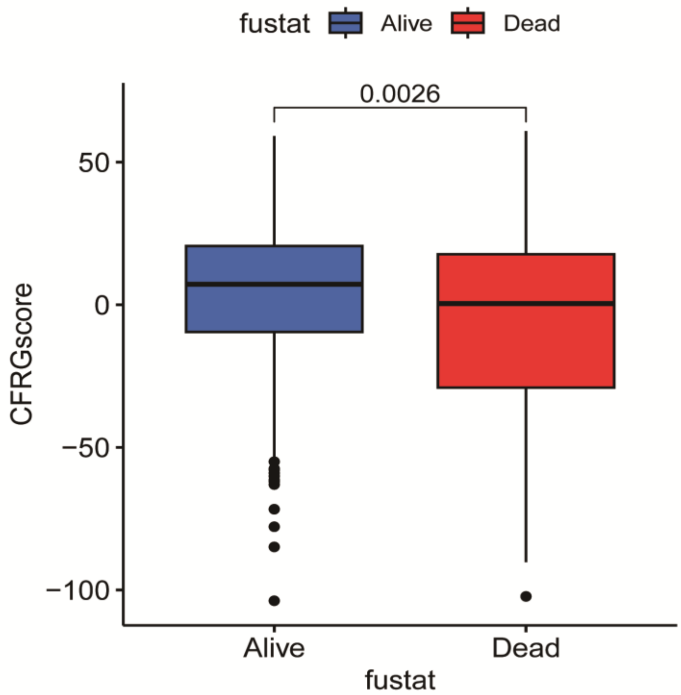

B

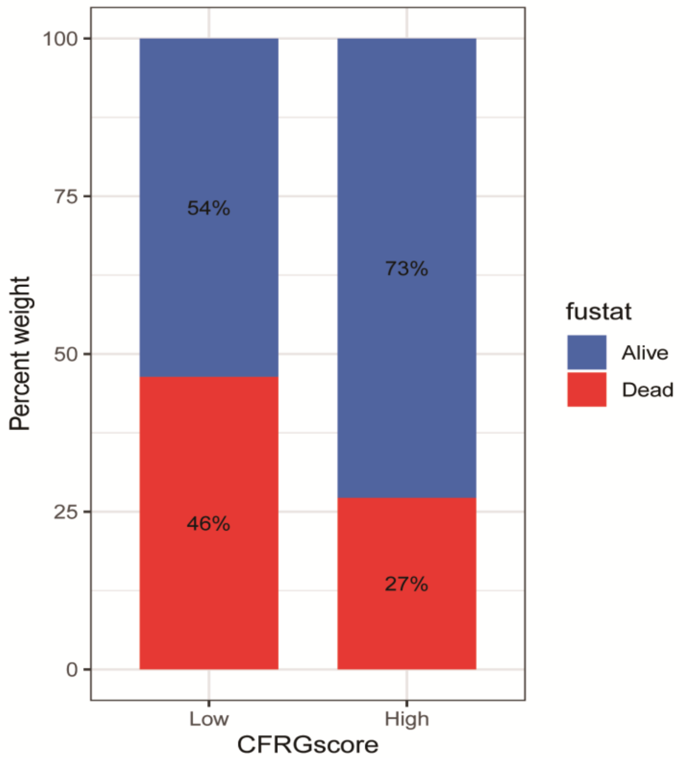

Fig S1 (A) Survival state between high- and low-CFRGscore groups. (B) The proportion of survival state between the two CFRGscore groups

Table. S1 The KM analysis and Univer COX analysis identified 97 genes related to prognosis

AURKA	1.216811	1.060499	1.396163	0.005153	0.000341
TYRO3	1.181938	1.031032	1.354932	0.016464	0.003533
TIMM9	1.77097	1.327182	2.363153	0.000103	2.04E-06
ABHD12	1.538221	1.196951	1.976793	0.000766	0.00033
TRIB3	1.223106	1.07767	1.388169	0.00182	1.72E-05
SUV39H1	1.358608	1.076056	1.715353	0.009991	0.001494
MAPK3	1.420786	1.103275	1.829672	0.006497	4.32E-05
ABCC1	1.292899	1.107446	1.509409	0.001146	0.000574
NDRG1	1.229089	1.085579	1.391571	0.001129	5.36E-05
SLC1A5	1.287696	1.156464	1.43382	4.01E-06	6.06E-09
TGFB1	1.19337	1.041814	1.366972	0.010738	0.000485
DNAJB6	1.813426	1.324892	2.482098	0.000202	1.55E-08
EZH2	1.47489	1.23575	1.760307	1.67E-05	1.22E-07
CREB3	1.332253	1.026028	1.729873	0.031335	0.003283
INTS2	1.366276	1.074055	1.738003	0.011028	0.000127
SLC38A1	1.253641	1.119695	1.40361	8.82E-05	3.65E-09
COPZ1	1.800924	1.302559	2.489965	0.000372	4.45E-06
CDCA3	1.399525	1.189395	1.646778	5.13E-05	7.44E-06
SLC39A7	1.390132	1.099459	1.757654	0.00592	0.000256
KIF20A	1.384935	1.200474	1.59774	7.99E-06	9.31E-12
ABCC5	1.590609	1.229865	2.057168	0.000405	6.15E-05
SLC1A4	1.340353	1.11636	1.609289	0.001691	3.88E-05
NCF2	1.201365	1.052846	1.370835	0.006433	0.000197
SLC2A1	1.422213	1.246224	1.623055	1.73E-07	1.54E-09
PRDX1	1.41256	1.134661	1.75852	0.002	9.47E-06
STMN1	1.35207	1.168223	1.56485	5.23E-05	1.86E-05
MYB	1.782289	1.251006	2.539198	0.001374	0.000196
MAPKAP1	1.422635	1.011964	1.999964	0.04252	0.007486
HELLS	1.299176	1.080247	1.562473	0.005439	0.00057
KDM3B	1.32964	1.049749	1.684158	0.018149	0.000207
KLHDC3	1.329243	1.034494	1.707972	0.026075	0.000413
PARP2	1.443165	1.083487	1.922242	0.012134	0.000577
MLLT1	1.396721	1.053155	1.852368	0.020368	9.37E-05
PPARG	1.261187	1.076891	1.477023	0.003989	3.32E-06
MYCN	1.329404	1.168983	1.511839	1.43E-05	0.000231
PHF21A	1.540922	1.216594	1.951711	0.000336	4.67E-06
DCAF7	1.412991	1.125135	1.774493	0.002936	0.000779
TXN	1.22243	1.004628	1.48745	0.044847	0.02646
ENPP2	1.120088	1.000858	1.253522	0.04828	0.003791
TFAP2A	1.292421	1.038039	1.609141	0.021802	5.77E-05
MTCH1	1.484314	1.136445	1.938667	0.003747	0.000287
RPTOR	1.425979	1.059151	1.919855	0.019351	0.000508
PGD	1.370203	1.150004	1.632565	0.000426	0.00013
FANCD2	1.386756	1.128534	1.704063	0.00187	0.000391
VLDLR	1.182639	1.01765	1.374377	0.028656	0.006588
SLC7A11	1.348337	1.181893	1.53822	8.75E-06	8.75E-09
BRPF1	1.515225	1.13375	2.025055	0.004981	6.08E-09
G6PD	1.298194	1.168466	1.442324	1.18E-06	1.54E-08
TOR2A	1.495547	1.08861	2.054603	0.012994	0.001013
SQSTM1	1.228565	1.060389	1.423414	0.006132	0.000487
PRKAA2	1.217143	1.057549	1.40082	0.00614	0.000291
ALB	0.899873	0.828352	0.97757	0.01253	7.24E-05
CDC25A	1.422129	1.198454	1.68755	5.50E-05	6.46E-06
VDAC2	1.567198	1.159303	2.11861	0.003489	0.001369
OTUB1	1.946082	1.434553	2.640012	1.88E-05	5.85E-06
MLST8	1.399794	1.029591	1.903109	0.03187	0.003168
NT5DC2	1.209697	1.071452	1.36578	0.002108	4.18E-07
RRM2	1.372522	1.187801	1.58597	1.76E-05	1.29E-06
CFL1	1.853916	1.411423	2.435134	9.14E-06	6.09E-06
MAP3K11	1.447844	1.05133	1.993906	0.023419	0.003002
HRAS	1.369786	1.107651	1.693959	0.003692	0.000294
[illegible]	[illegible]	[illegible]	[illegible]	[illegible]	[illegible]
